# Diverse distribution of Toxin-Antitoxin II systems in *Salmonella enterica* serovars

**DOI:** 10.1038/srep28759

**Published:** 2016-06-30

**Authors:** Andrea Di Cesare, Carmen Losasso, Lisa Barco, Ester M. Eckert, Daniele Conficoni, Giulia Sarasini, Gianluca Corno, Antonia Ricci

**Affiliations:** 1 Microbial Ecology Group, National Research Council – Institute of Ecosystem Study (CNR-ISE), Largo Tonolli 50,28822, Verbania, Italy; 2 Food Safety Department, Istituto Zooprofilattico Sperimentale delle Venezie, viale dell’Università 10, 35020, Legnaro, Italy; 3 Department Animal Medicine, Production and Health, University of Padua, viale dell’Università, 35020, Legnaro, Italy

## Abstract

Type II Toxin-Antitoxin systems (TAs), known for their presence in virulent and antibiotic resistant bacterial strains, were recently identified in *Salmonella enterica* isolates. However, the relationships between the presence of TAs (*ccd*AB and *vap*BC) and the epidemiological and genetic features of different non-typhoidal *Salmonella* serovars are largely unknown, reducing our understanding of the ecological success of different serovars. *Salmonella enterica* isolates from different sources, belonging to different serovars and epidemiologically unrelated according to ERIC profiles, were investigated for the presence of type II TAs, plasmid content, and antibiotic resistance. The results showed the ubiquitous presence of the *vap*BC gene in all the investigated *Salmonella* isolates, but a diverse distribution of *ccd*AB, which was detected in the most widespread *Salmonella* serovars, only. Analysis of the plasmid toxin *ccd*B translated sequence of four selected *Salmonella* isolates showed the presence of the amino acid substitution R99W, known to impede *in vitro* the lethal effect of CcdB toxin in the absence of its cognate antitoxin CcdA. These findings suggest a direct role of the TAs in promoting adaptability and persistence of the most prevalent *Salmonella* serovars, thus implying a wider eco-physiological role for these type II TAs.

*Salmonella* spp. are the second most frequent zoonotic agent in the European Union (EU)^1^ and represent a major challenge for animal health and food safety because of their high endemicity and morbidity rate, and of the difficulty in controlling the pathogen^2^. Salmonellosis is also the most common foodborne illness in the United States (US)^3^, causing the largest number of deaths and having the highest cost burden. Majowicz *et al*.^4^ estimated that 93.8 million cases of gastroenteritis due to *Salmonella* species occur globally each year, of which 80.3 million are foodborne, and which cause 155,000 deaths. In the EU, in 2013, 22.5% of all food-borne outbreaks were related to *Salmonella*, and 82,694 confirmed cases of human salmonellosis were reported^1^.

*Salmonella enterica* serovar Enteritidis and *Salmonella enterica* serovar Typhimurium (plus its monophasic variant *S*. 1,4,[5],12:i:-) are considered of paramount public health significance in the EU, as they accounted for 39.5%, 20.2%, and 8.6%, respectively, among all reported serovars in confirmed human infections in 2013^1^. The occurrence of *S*. 1,4,[5],12:i:- is constantly increasing over time throughout the EU^1^,^5^,^6^. US data from food-borne outbreaks related to human illness, collected over the period 2007–2011, confirmed that *S*. Enteritidis was the predominant serovar followed by *S*. Typhimurium^7^. In Italy, the picture is slightly different, since in the last few years *S*. 4,[5],12:i:- has become the predominant serovar responsible for human infection followed by *S*. Typhimurium, whereas *S*. Enteritidis has been displaying a marginal role in terms of public health^8^. In animals, non-motile *S.* Gallinarum is a host specific serovar, which causes great concern due to its high infectivity^9^.

There is much evidence suggesting that only a few *Salmonella* serovars are responsible for the vast majority of human infection cases, but, at the same time, it is unclear why these common serovars might have greater ecological success. A combination of different factors specific to each serovar, such as the presence of virulence plasmids, the cell surface structure, the presence of flagellin genes and the *Salmonella* pathogenicity islands, can be associated with the severity of human *Salmonella* infections^10^. Studies conducted to date have revealed that host range and virulence of *Salmonella* serovars are evolutionarily related to gene acquisition by horizontal transfer of mobile elements and by the loss of genes or gene functionality^11^.

Following these considerations, great interest exists in reducing the *Salmonella* impact on human and animal health by lowering its prevalence along the food chain through the implementation of control measures from farm to fork.

A deeper knowledge of the eco-evolutionary mechanisms enabling some *Salmonella* serovars to be widespread will lead to a better understanding of the epidemiology of virulence and finally to the design of more efficient control actions.

Widespread and virulent *Salmonella* serovars, such as *S*. Typhimurium and *S*. 4,[5],12:i:-, frequently exhibit a higher frequency of multidrug resistance phenotype than rare and less virulent serovars^5^,^12^,^13^. Furthermore, the genes for antibiotic resistance often occur on incompatible plasmids^14^. Many of these plasmids encode addiction systems, and harbour toxin-antitoxin (TA) factors, thus promoting post-segregation killing to maintain themselves^15^. The permanence of these specific plasmids in the bacterial population is promoted by the action of TA systems, regardless of other selective pressures^15^,^16^.

In particular, the type II TA modules, a pair of genes encoding a protein that interferes with basic cell metabolism (the toxin) and its antagonist (the antitoxin), are generally considered as genetic elements involved in bacterial stress response activities *via* a persistence mechanism^17^. Furthermore, the involvement of TA systems in a wide range of biological functions including growth control, defense against phages, and biofilm formation has been highlighted by recent research^18^,^19^,^20^.

Analysis of the *Salmonella* genome revealed the presence of 24 *Salmonella* TA loci^21^,^22^ accounting for 5 type I and 19 type II TA modules, most of which showed a narrow distribution restricted to pathogenic *Salmonella enterica*, and which were missing in poor pathogenic specie *S. bongori*^21^. This observation led Lobato-Màrquez and co-workers to hypothesize an active role for TA modules in enhancing the fitness of pathogenic *Salmonella* inside eukaryotic cells^21^.

The current study is focused on two type II TA systems, *vap*BC and *ccd*AB, associated with virulence in *Salmonella* and for which no clear evidence on their diffusion among virulent and widespread *Salmonella* serovars exists to date.

*Vap*BC (virulence associated protein) locus, originally called *vag*CD^23^ was identified for the first time on a *S.* Dublin virulence plasmid^24^. VapC was classified as a PIN domain ribonuclease, and originally reported to inhibit translation by cleaving the initiator tRNAfMet^25^.

*Ccd*AB (coupled cell division locus) has been described as involved in plasmid maintenance^23^. In the case of *S.* Typhimurium, it was mapped on the virulence plasmid pSLT^21^. The *ccd*B gene encodes a 101 amino acid residue protein able both to bind free DNA gyrase and the DNA-gyrase complexes, thus inhibiting DNA gyrase activity and/or trapping the gyrase-DNA cleavable complex; this causes DNA lesions, leading to the formation of lethal double strand DNA breaks. Moreover, the DNA gyrase inhibition, in the context of the gyrase-DNA complex, is suppressed by mutations occurring in the last three amino acid residues of CcdB that, in turn, inactivate the killing phenotype of CcdB^26^,^27^,^28^,^29^,^30^,^31^,^32^.

This study aimed to further our understanding of the eco-physiological role for these type II TAs, on a variety of isolates belonging to different serovars, including serovars which are commonly associated with human infections (*S*. Typhimurium, *S*. Enteritidis, *S*. 4,[5],12:i), species-specific serovars such as *S*. Gallinarum, and some other serovars, which have been rarely or never related to documented cases of infection, neither in animals nor in humans (*S*. Tennessee, *S*. Lawndale and *S*. Alachua). The presence of type II TAs in the different *Salmonella* serovars was thus examined in relation to the isolates’ sources, specific antibiotic resistance, and plasmid profile.

## Results

### Isolates selection

**Figure 1:**
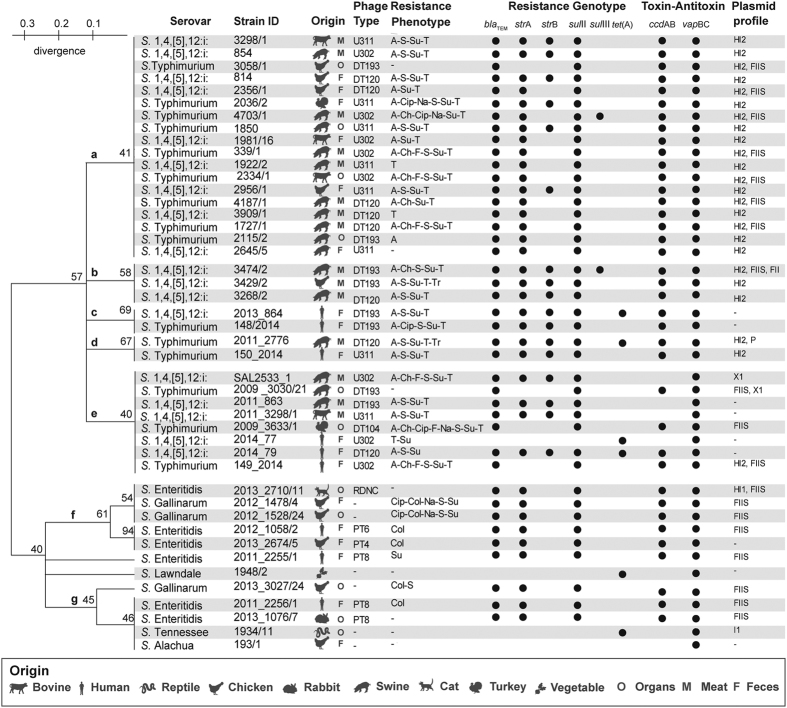
Phenotypic and molecular characterization of the investigated *Salmonella* strains. The dendrogram shows the similarity among the isolates tested by ERIC-PCR resulting in seven clusters: a–g. The source of isolation, phage-type, antibiotic resistance profile, toxin-antitoxin presence and plasmid profile of the isolates are listed. Ampicillin (A), chloramphenicol (Ch), ciprofloxacin (Cip), colistin (Col), florfenicol (F), nalidixic acid (Na), streptomycin (S), sulfamethoxazole (Su), tetracycline (T), and trimethoprim (Tr).

The 45 isolates (Fig. 1) comprised *S*. 4,[5],12:i:- (n = 18), *S*. Typhimurium (n = 15), *S*. Enteritidis (n = 5), *S*. Gallinarum (n = 3) and one each of *S*. Tennessee, *S*. Lawndale and *S*. Alachua. The last three isolates, serovars: Tennessee, Lawndale and Alachua, have been only rarely isolated from humans; thus, they were included in the study as a comparison with the other widespread and prevalent serovars.

*S*. Lawdale and *S*. Alachua, to our knowledge, have never been involved in outbreaks. *S*. Tennessee was responsible for a few cases of infections associated with powdered milk products and infant formula^33^ or peanut butter^34^ in the USA and Canada, respectively.

The sources of the *Salmonella* isolates were: 15 from swine, 11 from chicken, 4 from bovine, 2 from turkey, 9 from humans and the remaining 4 from other origins (vegetables, reptile, cat and rabbit).

*S*. Typhimurium and *S*. 4,[5],12:i:- isolates were selected as they belonged to the five most common phage-types isolated in Italy (DT193, U311, DT120, U302 and DT104). *S*. Enteritidis isolates displayed the phage-types: PT4, PT6, PT8 and RDNC (Fig. 1).

### ERIC-PCR typing profiles

The 45 examined isolates showed genetic diversity. The isolates were grouped into two major clusters having less than 70% sample match similarity. The first cluster included all the *S*. Typhimurium and *S*. 4,[5],12:i:- isolates, which are known to be two closely related serovars^35^, whereas the second cluster included isolates of *S*. Enteritidis, *S*. Gallinarum and the rare serovars, *S*. Tennessee, *S*. Lawndale and *S*. Alachua (Fig. 1). Different sub-groups were identified within each of the two principal clusters. *S*. Typhimurium and *S*. 4,[5],12:i:- were grouped into five sub-clusters, while serovars belonging to the second cluster were assorted into two sub-clusters, irrespective of their sources of isolation. One isolate each of *S*. Gallinarum and *S*. Enteritidis did not sub-cluster with any other *Salmonella* isolates (Fig. 1). The rare serovars *S*. Tennessee and Alachua were sub-clustered together with two *S*. Enteritidis isolates, while *S*. Lawndale was found to be outside of any cluster (Fig. 1). In some cases, isolates belonging to the same serovar were grouped into different sub-clusters, and some sub-clusters included isolates belonging to different serovars.

### Antimicrobial Susceptibility Assessment

The phenotypic resistances of the *Salmonella* isolates to antimicrobials are shown in Fig. 1, and reveal various patterns of resistance. Specific resistances to ampicillin (A), trimethoprim (Tr), florfenicol (F), chloramphenicol (Ch) and tetracycline (T) were found exclusively among *S*. Typhimurium and *S*. 4,[5],12:i:-, which were also the serovars most frequently resistant to streptomycin (S), and sulfamethoxazole (Su). Four out of the six *S*. Enteritidis isolates were resistant only to Su (1 isolate) or Colistin (Col) (3 isolates), with the latter resistance also being shown by all the *S*. Gallinarum isolates. Resistance to quinolones and fluoroquinolones, nalidixic acid (Na) and ciprofloxacin (Cip), were displayed only by *S*. Typhimurium (4 out of 15 isolates) and *S*. Gallinarum (2 out of 3). The remaining serovars, *S*. Tennessee, *S*. Alachua and *S*. Lawndale were pansusceptible to all antimicrobials examined.

Moreover, half of the *S*. 4,[5],12:i:- isolates displayed a typical profile of tetra-resistance to A, S, Su and T. Eleven *S*. Typhimurium isolates were also resistant to these four antimicrobials, although they generally displayed resistances to other antimicrobials as well.

In order to get deeper insight into the transferability of the antimicrobial resistance patterns displayed by the selected isolates, molecular screening for the most common resistance genes (ARGs) was performed. The ARGs, *bla*TEM, *str*A and *sul*II, were commonly associated to different extents with serovars Typhimurium, 4,[5],12:i:-, Enteritidis and Gallinarum. In addition, some *S*. Typhimurium and *S*. 4,[5],12:i:- isolates contained *str*B and *sul*II genes (Fig. 1). In contrast, DNA belonging to *S*. Tennessee and *S*. Lawndale displayed *tet*(A), only. The T resistance gene (*tet*(A)) was also present in one *S*. Typhimurium and three *S*. 4,[5],12:i:- isolates. Finally, none of the examined genes was harbored by the *S*. Alachua isolate. The antimicrobial resistance genes *qnr*A, *qnr*S and *bla*SHV were not detected in any of the examined isolates.

### Inc-plasmid molecular typing

PCR-based replicon typing was applied to type the incompatibility (Inc) plasmids harbored by the investigated isolates. A variety of Inc-plasmids were present in *S*. 4,[5],12:i:- and in *S*. Typhimurium isolates, as the former harbored HI2, FIIS, FII and XI and the latter HI2, FIIS and P plasmids (Fig. 1). In particular, HI2 plasmids were harbored by all *S*. 4,[5],12:i:- and *S*. Typhimurium strains belonging to the subclusters a, b and d. Conversely, serovars Enteritidis and Gallinarum displayed only FIIS plasmids. *S*. Lawndale and *S*. Alachua did not display any Inc-plasmid and *S*. Tennessee was the only isolate found to harbor I1 plasmids. Statistical analysis indicated a significant association between the presence of FIIS plasmids and S*almonella* serovars. The occurrence of this plasmid was significantly lower in the Typhimurium and 4,[5],12:i:- serovars compared to the other investigated serovars (*p* < 0.05).

### Screening for the presence of type II TA modules

Different patterns of presence of the *vap*BC and *ccd*AB TA modules characterized the DNA extracted from the *Salmonella* isolates. *vap*BC was constitutively found in every examined isolate, while *ccd*AB was present only in *S*. Typhimurium, *S*. Enteritidis and *S*. Gallinarum serovars, but was absent in the rare serovars Tennessee, Lawndale and Alachua (Fig. 1). *S*. 4,[5],12:i:- strains harbored *ccd*AB with the exception of 4 strains (SAL2533_1, 2011_863, 2011_32981 and 2014_77), all belonging to sub-group e (Fig. 1).

### CcdB sequence analysis

**Figure 2:**
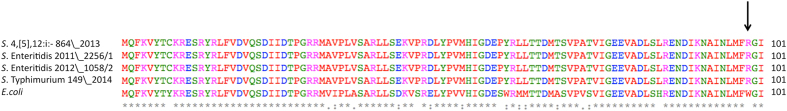
Alignment of the aminoacidic CcdB sequence. The arrow indicates the R99W mutation.

Comparison between the CcdB protein sequence of the isolates *S*. 4,[5],12:i:- 864_2013, *S*. Enteritidis 2011_2256/1, *S*. Enteritidis 2012_1058/2 and *S*. Typhimurium 149_2014 and of *E. coli*^31^ revealed numerous amino acid substitutions in these *Salmonella* isolates (Fig. 2) among which the R99W mutation was present in all examined *Salmonella*. Residue W99 is reported to be crucial for CcdB toxicity in *E. coli*^31^.

## Discussion

In this study, the spread of type II TAs, in relation to source, specific antibiotic resistance, and plasmid profile was studied through analysis of 45 *Salmonella* isolates belonging to highly pathogenic serovars, both for humans and animals, as well as serovars which have been rarely associated with infection episodes.

The isolates were tested for their heterogeneity by means of ERIC-PCR subtyping. In fact, by complementing phenotypic data, ERIC-PCR has been demonstrated as allowing rapid and cost effective exploration of the genetic relatedness among any one *Salmonella* serovar^36^ and thus being a rapid tool to broadly investigate genetic relatedness among isolates.

The close relationship between *S*. Typhimurium and S. 4,[5],12:i:- was confirmed; in consequence, all isolates belonging to these two serovars were included in the same cluster and were clearly differentiated from all other investigated serovars. The other serovars studied were grouped in a second cluster, including two serologically related serovars (i.e. *S*. Enteritidis and *S*. Gallinarum), and other serovars that did not share somatic and/or flagellar antigens. Within this cluster, isolates of the same serovar were then included in different sub-groups and each sub-group included isolates belonging to different serovars. The frequency of antimicrobial resistance phenotypes was higher among *S*. Typhimurium and *S*. 4,[5],12:i:- isolates in comparison to the other investigated serovars, with tetra-resistance “ASSuT” being the predominant multiple drug resistance profile in these two serovars.

The presence of ASSuT has become important in Italy since the year 2000, being increasingly detected both in *S*. Typhimurium and in *S*. 4,[5],12:i:-6,37. Moreover, *S*. Typhimurium isolates are more frequently resistant to F and Ch, while these resistances appear to be less common in *S*. 4,[5],12:i-.

A low level of antimicrobial resistance, as previously described by other authors^38^,^39^, was found for *S*. Enteritidis, even though it is known to be one of the most common serovars in human infections.

To investigate the plasmid content and thus the potential mobilization of ARGs, *Salmonella* isolates were examined for the presence of the major Inc plasmid groups by replicon type analysis. The results showed the presence of a heterogeneous panel of plasmids in the epidemiologically unrelated *S*. Typhimurium and *S*. 4,[5],12:i:- isolates. The observed variability of plasmid content among the examined isolates highlights the diversity of the *Salmonella* serovars. IncHI2 and IncFIIS were the most common plasmid groups among the investigated isolates. Their widespread presence suggests that they might have a role in *Salmonella* acquiring and transferring genetic material. In fact, IncHI2 and IncFIIS replicons are common in *Enterobacteriaceae* and have been recently associated with highly disseminated *β*-lactamase genes in *E. coli* and *Salmonella* from food animals^40^,^41^,^42^.

IncHI2 plasmids belonging to the major IncHI group are usually associated with human pathogens and carry antibiotic and heavy metal resistance genes^43^. In the investigated isolates, IncHI2 plasmids were present only in *S*. Typhimurium and *S*. 4,[5],12:i:- belonging to three diverse sub-clusters thus suggesting a direct influence of the phylogeny of the strains in the acquisition/permanence of specific Inc plasmid groups.

No significant relationship between the presence of IncHI2 plasmids and any specific antibiotic resistance pattern was observed. Concerning IncF plasmids, they have been found only in the *Enterobacteriaceae* family^44^ as carriers of virulence and antibiotic resistance determinants^45^. Moreover, they display multiples alleles for each of the replicons FII, FIA, FIB and FIC. FIIS is a subgroup of the IncFII replicon which is strictly associated with *Salmonella* spp^44^. The occurrence of IncFIIS plasmids in *Salmonella* isolates of various serovars in our study highlights their tendency to spread. Moreover, the absence of these replicons in the rare *Salmonella* serotypes Tennessee, Alachua and Lawndale evokes their involvement in the epidemiology of *Salmonella*^14^. However, the presence of this plasmid was significantly associated with serovars other than Typhimurium and 4,[5],12:i:- suggesting its diverse distribution may be strictly serovar dependent. Other Inc plasmid groups (i.e. IncHI2, FII, X1, I1 and P) were only sporadically present in the examined isolates, suggesting a limited but somehow positive participation in antibiotic resistance spread.

*S. enterica* evolved as a pathogen through a sequential order of events starting with the acquisition of genetic material by horizontal gene transfer, for example, pathogenicity islands and cargo genes on prophages and insertion elements. *Salmonella* evolution has continued through the acquisition of pseudogenes, which also led to host adaptation of a number of *Salmonella* serovars^9^. These genetic differences contributed to variations in terms of host range and virulence among different serovars^46^ and could explain the variation in pathogenicity among the serovars. As a model pathogen and worldwide cause of disease in both humans and animals, *Salmonella* is an important focus of novel research into myriad aspects of pathogenesis, including its capability to rapidly adapt to and survive ever-changing environments. This ability is essential for a foodborne pathogen that upon ingestion by a host, and in a short period of time, will switch from a free-living state in the contaminated food to a parasitic existence in a host.

Type II TA modules support bacterial adaptability in response to unfavorable environmental conditions and contribute to the generation of non-growing cells in response to stress, thus self-protecting bacteria by making them less sensitive to harmful environments^17^,^47^,^48^,^49^. These modules abound in bacterial pathogens and are thought to be associated with pathogenicity in epidemic bacteria, although this hypothesis is still debated^50^. TA operons are likely acquired by bacteria through horizontal gene transfer and then positively selected because of the advantage given to pathogens in favorable ecological settings^20^.

*Vap*BC and *ccd*AB, could be associated with virulence because of their possible localization on the virulence plasmid (pSLT) in the case of *S*. Typhimurium.

A closer look at the distribution of *vap*BC and *ccd*AB in the serovars examined in the present study revealed the constitutive occurrence of *vap*BC in all isolates, coupled with the more limited presence of the *ccd*AB locus. This locus was mainly restricted to widespread isolates, and was missing in the three isolates of serovars which only rarely have been linked to human salmonellosis. This finding confirms the observations made by De la Cruz and colleagues (2013)^51^, who found virulent serovars of *S. enterica* displaying multiple TA systems, in contrast to less-pathogenic isolates which harbored no or low numbers of TA modules.

*S*. Enteritidis, *S*. Typhimurium, and *S*. 4,[5],12:i:- are the three most prevalent serovars in Europe^1^; thus, the widespread occurrence of both the TA operons among their genomes, as demonstrated by the present study, might represent a selective advantage supporting their successful diffusion.

The complete sequencing of *S*. Typhimurium strain LT2 revealed the presence of differences in the CcdB codons compared to its *E. coli* active ortholog^22^. In particular, one of the differences was the R99W amino acid substitution, known to compromise *in vitro* the lethal effect of CcdB, when expressed in *S*. Typhimurium in the absence of its cognate antitoxin CcdA^21^,^31^. Our results confirmed the presence of the same substitution in the *Salmonella* isolates examined, as an Arginine was coded in the 99th amino acid position. This suggests the possibility that in *Salmonella*, plasmid toxin CcdB could play a different role than that of post-segregational killing, and instead, it could be connected to the activation of persistent phenotypes. This could represent an advantage for the most prevalent *Salmonella* serovars to adapt to different ecological niches and to resist changing environmental conditions.

## Methods

### *Salmonella* isolates

The *Salmonella* isolates studied are reported in Fig. 1. *Salmonella* isolates from animals and foodstuffs were collected by the OIE Reference Laboratory for Salmonellosis (Istituto Zooprofilattico Sperimentale delle Venezie, Legnaro, Italy) over the period 2010–2015. Human-derived *Salmonella* isolates were kindly provided by Dr. Ida Luzzi (Istituto Superiore di Sanità, Rome, Italy).

*Salmonella* isolates were grouped on the basis of their importance for public health and according to the following criteria: (i) originating from animal, foodstuff or human sources; (ii) representative of different animals species and foodstuff type; (iii) belonging to invasive, virulent and widespread serovars such as Enteritidis, Typhimurium, 4,[5],12:i:- or Gallinarum; (iv) representative of different phage types. Isolates were selected in order to avoid those which could be epidemiologically related.

All isolates were serotyped according to the Kauffmann-White scheme using the traditional slide agglutination method, combined with a previously described PCR protocol to differentiate *S*. Typhimurium and *S*. 4,[5],12:i:-52.

*S*. Enteritidis, *S*. Typhimurium and *S*. 4,[5],12:i:-. were phage typed using the protocols^53^,^54^ and following the interpretative guidelines set out for *S*. Typhimurium and *S*. Enteritidis by the International Federation for Enteric Phage Typing (IFEPT, Laboratory of Enteric Pathogens, Health Protection Agency, Colindale, London, UK).

All isolates were processed both for genomic and plasmid DNA extraction. Total DNA was extracted by the classical boiling method, while the plasmid DNA was obtained from pelleted cells using the QIAprep Spin Miniprep Kit (Qiagen) and by applying the modifications to the protocols required for the isolation of high and low plasmid copy-number as described by the supplier.

### ERIC-PCR typing

ERIC-PCR was performed as described by Fendri *et al*.^36^. Briefly, PCRs were prepared by adding 2 *μ*l of DNA template to a reaction mix containing 12.5 *μ*l of Go-Taq Green Master Mix (Promega) and 1 *μ*M of each primer; the reaction volume was made up to 25 *μ*l with autoclaved and filtered Milli Q water (Millipore, Billerica, MA, USA). PCRs were carried out using a My Cycler thermocycler (Bio-Rad). Primer sequences ERIC-1R (ATGTAAGCTCCGGGGATTCAC) and ERIC2 (AAGTAAGTGACTGGGGTGAGCG) were designed by Versalovic and co-authors^55^. The ERIC-PCR profiles were obtained by electrophoresis of the different amplicons for 8 h at 40 V/cm, in 2% agarose Tris borate-EDTA (TBE) gel stained with Midori Green (Bulldog Bio). A 100 bp DNA Ladder H3 RTU (Nippon Genetics, Germany) was used as the PCR fragment size marker. Band profile similarities for the examined isolates were analyzed by Treecon software (Bioinformatics & Evolutionary Genomics, Belgium) and Dice coefficients were calculated. A bootstrap resampling process (100 reiterations) applying the neighbor joining (NJ) method was used to assess the robustness of each individual phylogenetic node. In order to cluster the isolates according to the unweighted pair group method of averages (UPGMA), a cut-off of 90% was set.

### Antimicrobial susceptibility testing

#### Phenotypic assay

The isolates were subcultured onto tryptone agar slants at 4 °C, transferred to 15 ml of Mueller-Hinton broth and incubated at 37 °C overnight. Antimicrobial susceptibility was tested by using a commercial microdilution test (Sensititre® *Salmonella* plate – EUMVS2) against a panel of 14 antimicrobials: ampicillin (A), cefotaxime (FOT), ceftazidime (TAZ), chloramphenicol (Ch), ciprofloxacin (Cip), colistin (Col), florfenicol (F), gentamicin (GEN), kanamycin (KAN); nalidixic acid (Na), streptomycin (S), sulfamethoxazole (Su), tetracycline (T), and trimethoprim (Tr) according to the manufacturer’s recommendations. The results were assessed after 24 h of incubation at 37 °C. The minimum inhibitory concentration (MIC) was defined as the lowest concentration of the antimicrobial that completely inhibited visible growth. The results were thus analyzed according to the cut-offs set by EUCAST (www.eucast.org).

#### Genotypic assay

Bacterial DNA extracted from each isolate was examined for the presence of a panel of ARGs conferring resistance to quinolones (*qnr*A and *qnr*S), *β*-lactams (*bla*TEM and *bla*SHV), S (*str*A and *str*B), T (*tet*(A)) and sulphonamides (*sul*II and *sul*III); the primer sequences are listed in Table 1 and the positive controls are listed in Table 2. Briefly, specific ARGs fragments were amplified by PCR essays, as previously described for ERIC-PCR except for the primer concentration (0.5 *μ*M) and the thermal profile (5 min at 95 °C, 30 cycles of 30 s at 95 °C, 30 s at specific annealing temperature (Table 1), 30 s at 72 °C and a final extension of 5 min at 72 °C).

### Plasmid molecular typing

DNA from *Salmonella* isolates was examined for the presence of the following incompatibility plasmid groups: Inc (A/C, HI1, HI2, I1, I2, L/M, N, K, B/O, W, P, T, U, R, Y, X1, X2, HIB-M, FIB-M, FIA, FIB, FIC, FII, FIIS, FIIk). Plasmid replicons of *Salmonella* isolates were detected and typed by PCR-based replicon typing (PBRT) as proposed by Villa *et al*.^44^, using the PBRT kit from DIATHEVA (DIATHEVA, Fano PU, Italy) according to the manufacturer’s instructions.

### Screening for the presence of type II TA modules

DNA of each isolate was examined by PCR for the presence of two *Salmonella* type II TAs: *vap*BC and *ccd*AB. Primers sequences vapB-f (TGAGYACCAGAGAACAACC), vapC-r (GAYGGAGCTGATACACATTC), ccdA-f (TTGCTGACGAVAACAGGAAC) and ccdB-r (TATGCAYCACCGGGTAAAG) were designed based on conserved regions of TA genes using *Clustal-XII* software (http://www.clustal.org/) for multiple alignment, *NetPrimer* software (http://www.premierbiosoft.com/netprimer/index.html) for the primer design and BLAST analysis in order to verify primer specificity. PCR assays were performed as described above for ARGs detection with the annealing temperatures of 55 °C for both *vap*BC and *ccd*AB genes.

### CcdB sequence analysis

A fragment of 632 bp of the CcdB toxin gene containing its entire coding sequence (CDS) from four *Salmonella* isolates (*S*. 4,[5],12:i:- 864_2013, *S*. Enteritidis 2011_2256/1, *S*. Enteritidis 2012_1058/2 and *S*. Typhimurium 149_2014) was amplified by PCR using the primers CCD_B fw (5′-TACGACCATGCAGAACGAAG-3′) and CCD_B rw (5′-CACTTCTGTACCACCGCAAA-3′), designed as previously described on the conserved region of textitS. Typhimurium strain SL1344 based on annotations and the sequence deposited in NCBI (entries NC_017720.1 (plasmid 1, pSLT) gene ID PSLT106). To achieve high fidelity gene amplifications, the Phusion® High-Fidelity DNA Polymerase (New England Biolabs Inc.) was used and the following thermal profile was applied: 30 s at 98 °C, 25 cycles of 15 s at 98 °C, 30 s at 53 °C, 30 s at 72 °C and a final extension of 5 min at 72 °C. Amplicons were then bidirectionally sequenced, assembled and manually corrected using DNA Baser Sequence Assembly Software (Heracle BioSoft, Romania). The sequences were deposited at the European Nucleotide Archive (ENA) with accession numbers LN897324-LN897327. The assembled sequences were translated to proteins using the *Translate* tool (ExPASy-Bioinformatic Resources Portal, http://web.expasy.org/translate/). Protein sequences were aligned by means of the *ClustalW2* tool (EMBL EBI, http://www.ebi.ac.uk/Tools/msa/clustalw2/) using the *E. coli* CcdB sequence as reference.

### Statistical analysis

The Pearson Chi Square test was used to evaluate possible associations between the presence of FIIS plasmids and *Salmonella* serovars (*S*. Typhimurium and *S*. 4,[5],12:i:- vs. other serovars). To verify whether a significant difference exists in the prevalence of FIIS plasmids among the investigated serovars, the z-test was used. P values less then 0.05 were considered significant. STATA 12 was used to analyze the data.

## Additional Information

**How to cite this article**: Di Cesare, A. *et al*. Diverse distribution of Toxin-Antitoxin II systems in *Salmonella enterica* serovars. *Sci. Rep.* **6**, 28759; doi: 10.1038/srep28759 (2016).

## Figures and Tables

**Table 1:** Primer pairs used to amplify antibiotic resistance genes.

Target Name	Antibiotic class	Primer sequence (5′-3′)	Amplicon size (bp)	Annealing temperature (°C)	Reference
*tet*(A)	Tetracycline	1-GCTACATCCTGCTTGCCTTC	210	56	56
		2-CATAGATCGCCGTGAAGAGG			
*bla*TEM	*β*-Lactams	1-TTCCTGTTTTTGCTCACCCAG	112	58	57
		2-CTCAAGGATCTTACCGCTGTTG			
*bla*shv	*β*-Lactams	1-CGCTTTCCCATGATGAGCACCTTT	110	58	58
		2-TCCTGCTGGCGATAGTGGATCTTT			
*str*A	Aminoglycosides	*1-TCAATCCCGACTTCTTACCG*	126	54	59
		2-CACCATGGCAAACAACCATA			
*str*B	Aminoglycosides	1-ATCGCTTTGCAGCTTTGTTT	143	54	59
		2-ATGATGCAGATCGCCATGTA			
*qnr*A	Quinolones	1-ATTTCTCACGCCAGGATTTG	159	54	60
		2-GCAGATCGGCATAGCTGAAG			
*qnr*S	Quinolones	1-GACGTGCTAACTTGCGTGAT	118	54	61
		2-TGGCATTGTTGGAAACTTG			
*sul*II	Sulphonamides	1-TCCGGTGGAGGCCGGTATCTGG	191	58	62
		2-CGGGAATGCCATCTGCCTTGAG			
*sul*III	Sulphonamides	1-TCCGTTCAGCGAATTGGTGCAG	128	60	62
		2-TTCGTTCACGCCTTACACCAGC			

**Table 2:** Positive control strains.

Bacterial strain	Antibiotic resistance gene (s)	Source
*Salmonella* 4764/5 (2009)	*bla*TEM, *sul*II, *str*A, *str*B	a
*Salmonella* 1279/2 (2011)	*bla*TEM, *tet*(A)	a
*Salmonella* 3674/2 (2011)	*tet*(A), *qnr*S	a
*E. coli* JM109	*qnr*A	b
*E. coli* DH5*α*	*sul*III	b
*Enterobacter cloacae* A1	*bla*shv	c

**a** From the collection of Department of Food Safety, Istituto Zooprofilattico Sperimentale delle Venezie, Legnaro, Italy; **b** From the collection of Department of Surface Waters-Research and Management, Eawag: Swiss Federal Institute of Aquatic Science and Technology, Kastanienbaum, Switzerland; **c** From the collection of Department of Infectious, Parasitic and Immune-Mediated Diseases, Istituto Superiore di Sanità, Rome, Italy.

## References

[b1] EFSA & ECDC. The European Union summary report on trends and sources of zoonoses, zoonotic agents and food-borne outbreaks in 2013. EFSA Journal 13, 3991–4156 (2015). 10.2903/j.efsa.2018.5500 PMC7009540 32625785

[b2] Castiglioni Tessari E. N. . Important aspects of *Salmonella* in the poultry industry and in public health. Salmonella - A Dangerous Foodborne Pathogen. InTech 1–27 (2012).

[b3] Lienau E. K. . Phylogenomic Analysis Identifies Gene Gains That Define *Salmonella enterica* Subspecies I. PLoS ONE 8, e76821–9 (2013). 24204679 10.1371/journal.pone.0076821 PMC3810377

[b4] Majowicz S. E. . The Global Burden of Nontyphoidal *Salmonella* Gastroenteritis. Clin infect dis 50, 882–889 (2010). 20158401 10.1086/650733

[b5] Hopkins K. . Multiresistant *Salmonella enterica* serovar 4,[5],12:i:- in Europe: a new pandemic strain? Eurosurveillance 15, 1–9 (2010). 20546690

[b6] Dionisi A. M. . Molecular characterization of multidrug-resistant strains of *Salmonella enterica* serotype Typhimurium and Monophasic variant (S. 4,[5],12:i:-) isolated from human infections in Italy. Foodb pathog dis 6, 711–717 (2009). 10.1089/fpd.2008.0240 19580448

[b7] Andino A. & Hanning I. *Salmonella enterica*: Survival, Colonization, and Virulence Differences among Serovars. The Scientific World J 2015, 1–16 (2015). 10.1155/2015/520179 PMC4310208 25664339

[b8] Barco L. . Ascertaining the relationship between *Salmonella* Typhimurium and *Salmonella* 4,[5],12:i:- by MLVA and inferring the sources of human salmonellosis due to the two serovars in Italy. Front Microb 6, 1–10 (2015). 10.3389/fmicb.2015.00301 PMC4415582 25983720

[b9] Barrow P. A. & Methner U. Salmonella in Domestic Animals. 2nd Edition (CABI International, 2013).

[b10] Campioni F., Bergamini A. M. M. & Falcão J. P. Genetic diversity, virulence genes and antimicrobial resistance of *Salmonella* Enteritidis isolated from food and humans over a 24-year period in Brazil. Food Microbiol 32, 254–264 (2012). 22986188 10.1016/j.fm.2012.06.008

[b11] Foley S. L., Johnson T. J., Ricke S. C., Nayak R. & Danzeisen J. *Salmonella* Pathogenicity and Host Adaptation in Chicken-Associated Serovars. Microbiol and Molec Biol Rev 77, 582–607 (2013). 24296573 10.1128/MMBR.00015-13 PMC3973385

[b12] Moreno Switt A. I., Soyer Y. & Warnick L. D. Emergence, Distribution, and Molecular and Phenotypic Characteristics of *Salmonella enterica* Serotype 4, 5, 12: i:–. Foodb Pathog and Dis 6, 407–415 (2009). 10.1089/fpd.2008.0213 PMC3186709 19292687

[b13] On Biological Hazards, E. P. Scientific Opinion on monitoring and assessment of the public health riskof “. EFSA Journal 8, 1–48 (2010).

[b14] Carattoli A. Resistance Plasmid Families in Enterobacteriaceae. Antimicrob Agents and Chemother 53, 2227–2238 (2009). 19307361 10.1128/AAC.01707-08 PMC2687249

[b15] Hayes F. Toxins-Antitoxins: Plasmid Maintenance, Programmed Cell Death, and Cell Cycle Arrest. Science 301, 1496–1499 (2003). 12970556 10.1126/science.1088157

[b16] Cooper T. F. & Heinemann J. A. Postsegregational killing does not increase plasmid stability but acts to mediate the exclusion of competing plasmids. In Pnas, vol. 97, 12643–12648 (2000). 11058151 10.1073/pnas.220077897 PMC18817

[b17] Maisonneuve E. & Gerdes K. Molecular Mechanisms Underlying Bacterial Persisters. Cell 157, 539–548 (2014). 24766804 10.1016/j.cell.2014.02.050

[b18] Ghafourian S., Raftari M., Sadeghifard N. & Sekawi Z. Toxin-antitoxin Systems: Classification, Biological Function and Application in Biotechnology. Curr issues in mol biol 16, 9–14 (2014). 23652423

[b19] Goeders N. & Van Melderen L. Toxin-Antitoxin Systems as Multilevel Interaction Systems. Toxins 6, 304–324 (2014). 24434905 10.3390/toxins6010304 PMC3920263

[b20] Guglielmini J. & Van Melderen L. Bacterial toxin-antitoxin systems. Mobil Genet Elements 1, 283–306 (2014). 10.4161/mge.18477 PMC3337138 22545240

[b21] Lobato-Márquez D., Moreno-Córdoba I., Figueroa V., Daz-Orejas R. & Garca-del Portillo F. Distinct type I and type II toxin-antitoxin modules control *Salmonella* lifestyle inside eukaryotic cells. Scien Rep 5, 9374–10 (2015). 10.1038/srep09374 PMC4366850 25792384

[b22] McClelland M. . Complete genome sequence of *Salmonella enterica* serovar Typhimurium LT2. Nature 413, 852–856 (2001). 11677609 10.1038/35101614

[b23] Mnif B. . Molecular characterization of addiction systems of plasmids encoding extended-spectrum beta-lactamases in *Escherichia coli*. J Antimicrob Chemother 65, 1599–1603 (2010). 20507859 10.1093/jac/dkq181

[b24] Pullinger G. D. & Lax A. J. A. *Salmonella* Dublin virulence plasmid locus that affects bacterial growth under nutrient-limited conditions. Molec Microb 6, 1631–1643 (1992). 10.1111/j.1365-2958.1992.tb00888.x 1495391

[b25] Winther K. S. & Gerdes K. Enteric virulence associated protein VapC inhibits translation by cleavage of initiator tRNA. Pnas 108, 7403–7407 (2011). 21502523 10.1073/pnas.1019587108 PMC3088637

[b26] Bahassi E. M. . Interactions of CcdB with DNA gyrase. Inactivation of Gyra, poisoning of the gyrase-DNA complex, and the antidote action of CcdA. J biol chem 274, 10936–10944 (1999). 10196173 10.1074/jbc.274.16.10936

[b27] Bahassi E. M., Salmon M. A., Melderen L., Bernard P. & Couturier M. F plasmid CcdB killer protein *ccd*B gene mutants coding for non-cytotoxic proteins which retain their regulatory functions. Molec Microbiol 15, 1031–1037 (1995). 7623659 10.1111/j.1365-2958.1995.tb02278.x

[b28] Bernard P. & Couturier M. Cell killing by the F plasmid CcdB protein involves poisoning of DNA-topoisomerase II complexes. J mol biol 226, 735–745 (1992). 1324324 10.1016/0022-2836(92)90629-x

[b29] Dao-Thi M.-H. . Crystallization of CcdB in complex with a GyrA fragment. Acta Crystallog Section D Biol Crystallog 60, 1132–1134 (2004). 10.1107/S0907444904007814 15159578

[b30] Kampranis S. C., Howells A. J. & Maxwell A. The interaction of DNA gyrase with the bacterial toxin CcdB: evidence for the existence of two gyrase-CcdB complexes. J mol biol 293, 733–744 (1999). 10543963 10.1006/jmbi.1999.3182

[b31] Loris R. . Crystal structure of CcdB, a topoisomerase poison from *E. coli*. J mol biol 285, 1667–1677 (1998). 9917404 10.1006/jmbi.1998.2395

[b32] Smith A. B. & Maxwell A. A strand-passage conformation of DNA gyrase is required to allow the bacterial toxin, CcdB, to access its binding site. Nucleic Acid Res 34, 4667–4676 (2006). 16963775 10.1093/nar/gkl636 PMC1635281

[b33] For Disease Control, C. & CDC, P. *Salmonella* serotype Tennessee in powdered milk products and infant formula–Canada and United States, 1993. MMWR Morbidity and mortality weekly report 42, 516–517 (1993). 8515742

[b34] Sheth A. N. . A national outbreak of *Salmonella* serotype Tennessee infections from contaminated peanut butter: a new food vehicle for salmonellosis in the United States. Clin infec dis 53, 356–362 (2011). 21810748 10.1093/cid/cir407

[b35] Ido N. . Characteristics of *Salmonella enterica* Serovar 4,[5],12:i:- as a Monophasic Variant of Serovar Typhimurium. PLoS ONE 9, e104380–8 (2014). 25093666 10.1371/journal.pone.0104380 PMC4122451

[b36] Fendri I. . Genetic diversity of food-isolated *Salmonella* strains through Pulsed Field Gel Electrophoresis (PFGE) and Enterobacterial Repetitive Intergenic Consensus (ERIC-PCR). PLoS ONE 8, e81315–e81315 (2012). 24312546 10.1371/journal.pone.0081315 PMC3849149

[b37] Lucarelli C. . Evidence for a second genomic island conferring multidrug resistance in a clonal group of strains of *Salmonella enterica* serovar Typhimurium and its monophasic variant circulating in Italy, Denmark, and the United Kingdom. J Clin Microbiol 48, 2103–2109 (2010). 20410351 10.1128/JCM.01371-09 PMC2884514

[b38] Yang S. J. . Antimicrobial resistance in *Salmonella enterica* serovars Enteritidis and Typhimurium isolated from animals in Korea: comparison of phenotypic and genotypic resistance characterization. Vet Microbiol 86, 295–301 (2002). 11955779 10.1016/s0378-1135(02)00009-3

[b39] Mezal E. H. . Isolation and molecular characterization of *Salmonella enterica* serovar Enteritidis from poultry house and clinical samples during 2010. Food Microbiol 38, 67–74 (2014). 24290628 10.1016/j.fm.2013.08.003

[b40] Hopkins K. L. . Replicon typing of plasmids carrying CTX-M or CMY beta-lactamases circulating among *Salmonella* and *Escherichia coli* isolates. Antimicrob Agents Chemother 50, 3203–3206 (2006). 16940132 10.1128/AAC.00149-06 PMC1563510

[b41] Garca-Fernández A. . Multilocus sequence typing of IncI1 plasmids carrying extended-spectrum beta-lactamases in *Escherichia coli* and *Salmonella* of human and animal origin. J of antimicrob chemother 61, 1229–1233 (2008). 18367460 10.1093/jac/dkn131

[b42] Moodley A. & Guardabassi L. Transmission of IncN Plasmids Carrying blaCTX-M-1 between Commensal *Escherichia coli* in Pigs and Farm Workers. Antimicrob Agents Chemother 53, 1709–1711 (2009). 19188380 10.1128/AAC.01014-08 PMC2663060

[b43] Gilmour M. W., Thomson N. R., Sanders M., Parkhill J. & Taylor D. E. The complete nucleotide sequence of the resistance plasmid R478: defining the backbone components of incompatibility group H conjugative plasmids through comparative genomics. Plasmid 52, 182–202 (2004). 15518875 10.1016/j.plasmid.2004.06.006

[b44] Villa L., Garcia-Fernandez A., Fortini D. & Carattoli A. Replicon sequence typing of IncF plasmids carrying virulence and resistance determinants. J Antimicrob Chemother 65, 2518–2529 (2010). 20935300 10.1093/jac/dkq347

[b45] Johnson T. J. & Nolan L. K. Pathogenomics of the Virulence Plasmids of *Escherichia coli*. Microbiol and Mol Biol Rev 73, 750–774 (2009). 19946140 10.1128/MMBR.00015-09 PMC2786578

[b46] Matthews T. D. . Genomic Comparison of the Closely-Related *Salmonella enterica* Serovars Enteritidis, Dublin and Gallinarum. PLoS ONE 10, e0126883–18 (2015). 26039056 10.1371/journal.pone.0126883 PMC4454671

[b47] Yamaguchi Y. & Inouye M. Regulation of growth and death in *Escherichia coli* by toxin–antitoxin systems. Nature Rev: Microbiology 9, 779–790 (2011). 21927020 10.1038/nrmicro2651

[b48] Yamaguchi Y., Park J.-H. & Inouye M. Toxin-Antitoxin Systems in Bacteria and Archaea. Annual Rev of Genet 45, 61–79 (2011). 22060041 10.1146/annurev-genet-110410-132412

[b49] Wen Y., Behiels E. & Devreese B. Toxin-Antitoxin systems: their role in persistence, biofilm formation, and pathogenicity. Pathog and Dis 70, 240–249 (2014). 10.1111/2049-632X.12145 24478112

[b50] Georgiades K. & Raoult D. Genomes of the Most Dangerous Epidemic Bacteria Have a Virulence Repertoire Characterized by Fewer Genes but More Toxin-Antitoxin Modules. PLoS ONE 6, e17962–10 (2011). 21437250 10.1371/journal.pone.0017962 PMC3060909

[b51] De la Cruz M. A. . A toxin-antitoxin module of *Salmonella* promotes virulence in mice. PLoS Path 9, e1003827–e1003827 (2012). 10.1371/journal.ppat.1003827 PMC3868539 24385907

[b52] Barco L. . A rapid and sensitive method to identify and differentiate *Salmonella enterica* serotype Typhimurium and *Salmonella enterica* serotype 4,[5],12:i:- by combining traditional serotyping and multiplex polymerase chain reaction. Foodb pathogens dis 8, 741–743 (2011). 10.1089/fpd.2010.0776 PMC3117292 21247297

[b53] Anderson E. S., Ward L. R., Saxe M. J. & de Sa J. D. Bacteriophage-typing designations of *Salmonella* Typhimurium. J Hyg 78, 297–300 (1977). 321679 10.1017/s0022172400056187 PMC2129838

[b54] Ward L. R., de Sa J. D. & Rowe B. A phage-typing scheme for *Salmonella* Enteritidis. Epidem Infect 99, 291–294 (1987). 10.1017/s0950268800067765 PMC2249269 3315705

[b55] Versalovic J., Koeuth T. & Lupski J. R. Distribution of repetitive DNA sequences in eubacteria and application to fingerprinting of bacterial genomes. Nucleic Acids Res 19, 6823–6831 (1991). 1762913 10.1093/nar/19.24.6823 PMC329316

[b56] Ng L. K., Martin I., Alfa M. & Mulvey M. Multiplex PCR for the detection of tetracycline resistant genes. Mol and Cell Probes 15, 209–215 (2000). 10.1006/mcpr.2001.0363 11513555

[b57] Bibbal D. . Impact of Three Ampicillin Dosage Regimens on Selection of Ampicillin Resistance in Enterobacteriaceae and Excretion of *bla*TEM Genes in Swine Feces. Appl Environ Microbiol 73, 4785–4790 (2007). 17557857 10.1128/AEM.00252-07 PMC1951005

[b58] Xi C. . Prevalence of Antibiotic Resistance in Drinking Water Treatment and Distribution Systems. Appl Environmenl Microbiol 75, 5714–5718 (2009). 10.1128/AEM.00382-09 PMC2737933 19581476

[b59] Walsh F. . Real-time PCR methods for quantitative monitoring of streptomycin and tetracycline resistance genes in agricultural ecosystems. J Microbiol Meth 86, 150–155 (2011). 10.1016/j.mimet.2011.04.011 21549164

[b60] Marti E., Jofre J. & Balcazar J. L. Prevalence of Antibiotic Resistance Genes and Bacterial Community Composition in a River Influenced by a Wastewater Treatment Plant. PLoS ONE 8, e78906–8 (2013). 24205347 10.1371/journal.pone.0078906 PMC3808343

[b61] Marti E. & Balcazar J. L. Real-Time PCR Assays for Quantification of qnr Genes in Environmental Water Samples and Chicken Feces. Appl Env Microbiol 79, 1743–1745 (2013). 23275512 10.1128/AEM.03409-12 PMC3591933

[b62] Pei R., Kim S.-C., Carlson K. H. & Pruden A. Effect of River Landscape on the sediment concentrations of antibiotics and corresponding antibiotic resistance genes (ARG). Water Res 40, 2427–2435 (2006). 16753197 10.1016/j.watres.2006.04.017

